# Psoriasis severity classification based on adaptive multi-scale features for multi-severity disease

**DOI:** 10.1038/s41598-023-44478-9

**Published:** 2023-10-13

**Authors:** Cho-I. Moon, Jiwon Lee, Yoo Sang Baek, Onesok Lee

**Affiliations:** 1 Department of Software Convergence, Graduate School, Soonchunhyang University, 22, Soonchunhyang-ro, Asan, Chungnam-do 31538 Republic of Korea; 2 Department of Dermatology, Guro Hospital, Korea University College of Medicine, Seoul, 08308 Republic of Korea; 3 Department of Medical IT Engineering, College of Medical Sciences, Soonchunhyang University, 22, Soonchunhyang-ro, Asan, Chungnam-do 31538 Republic of Korea

**Keywords:** Health care, Medical research

## Abstract

Psoriasis is a skin disease that causes lesions of various sizes across the body and can persist for years with cyclic deterioration and improvement. During treatment, and a multiple-severity disease, with irregular severity within the observation area may be found. The current psoriasis evaluation is based on the subjective evaluation criteria of the clinician using the psoriasis area and severity index (PASI). We proposed a novel psoriasis evaluation method that detects representative regions as evaluation criteria, and extracts severity features to improve the evaluation performance of various types of psoriasis, including multiple-severity diseases. We generated multiple-severity disease images using CutMix and proposed a hierarchical multi-scale deformable attention module (MS-DAM) that can adaptively detect representative regions of irregular and complex patterns in multiple-severity disease analyses. EfficientNet B1 with MS-DAM exhibited the best classification performance with an F1-score of 0.93. Compared with the performance of the six existing self-attention methods, the proposed MS-DAM showed more than 5% higher accuracy than that of multiscale channel attention module (MS-CAM). Using the gradient-weighted activation mapping method, we confirmed that the proposed method works at par with human visual perception. We performed a more objective, effective, and accurate analysis of psoriasis severity using the proposed method.

## Introduction

Psoriasis is a skin disease characterized by erythematous plaques of various sizes. Lesions are typically covered by silvery-white scales, with relatively clear boundaries^1,2^. Histologically, it is characterized by epidermal hyperplasia. Psoriasis can occur all over the body and commonly affects the scalp, elbows, nails, buttocks, knees, and shins. Additionally, it exhibits the characteristic of appearing symmetrically. Psoriasis is a chronic inflammatory disease that can persist for many years or decades with cyclic deterioration and improvement. It is difficult to be completely healed and is classified as a chronic intractable skin disease. Therefore, it is very important not only to diagnose skin diseases such as psoriasis at an early stage but also to accurately evaluate the current disease state.

Currently, clinical trials and studies use the psoriasis area severity index (PASI) to evaluate the severity of psoriasis^3,4^. In some countries, including the Republic of Korea, financial benefits such as health reimbursement are provided to patients with chronically severe psoriasis lasting more than 6 months to reduce treatment costs. The PASI score is used as a reimbursement criterion for biologics in severe psoriasis before and after treatment of the disease. The severity of psoriasis is assessed through the PASI score, which is calculated by quantifying the percentage of the affected area and its severity (erythema, thickness, and scale) in each body region (head, arms, legs, and trunk). To determine the PASI score of the disease, clinicians perform visual inspection and palpation and gather subjective statements regarding the patient^5,6^. Psoriasis is characterized by dynamic changes, in which lesions occur or disappear during treatment. Multiple-severity diseases are observed with these dynamic changes. Multiple-severity diseases have several areas of observation with different severities or one large disease with irregular severities. Currently, in multiple-severity diseases, clinicians subjectively select the representative regions, which are used as evaluation criteria, to assess the severity score. Thus, disease evaluation relies on a subjective interpretation based on the proficiency and experience of the clinician, resulting in deviations in the evaluation results. Therefore, to objectify and quantify psoriasis evaluation, a new method that considers both the dynamic features of severe psoriasis and the clinician’s perspective is required.

Many studies have been conducted using skin erythema and hydration meters^7^, traditional conventional methods, and state-of-the-art (SOTA) machine learning and deep learning technologies to objectify the PASI score, which plays an important role in psoriasis diagnosis and treatment evaluation. First, erythema and scaling, which are psoriasis lesions, have distinct color differences according to severity. Many existing studies have performed disease analyses using the color features of the lesions. Clustering and support vector machines (SVM) are the most basic and widely used skin disease classification methods^8–10^. One study applied Gaussian mixture model-based clustering to psoriasis lesions to identify the affected areas and segmented the psoriatic plaques based on the color features of erythema and scaling^11^. Another study classified the abnormal redness by applying an SVM to normal and psoriatic skin images^5^. In another example, a study helped classify the psoriasis severity by evaluating erythema, which is one of the PASI parameters, by mapping skin lesion images into the CIE LAB color space^12^. Accurately determining the area of psoriasis based on the distinct color difference between erythema and scaling, which are typical characteristics of psoriasis, can make a major contribution to the severity assessment.

Recently, emerging artificial neural network-based deep learning models have been used to address various challenges in image detection, segmentation, and classification. Many scholars have discussed whether these methods can be applied to the analysis of medical images. Convolutional neural network (CNN)-based models automatically extract unchanging features from input images and interpret pixels directly from image contexts^13–15^. One study performed binary classification of the presence or absence of psoriasis based on contextual information in local psoriasis images using a deep neural network^16^. Another study achieved increased computational efficiency by empirically removing specific connections between convolutional layers and fully connected layers in the VGG-16 architecture and classified the severity of psoriasis into four stages^17^. A previous study compared the segmentation results of the SLIC superpixels method and the U-Net model for the psoriasis area and improved the severity classification performance by extracting the color features of erythema and scaling^18^. Another study evaluated the severity of psoriasis in two input images by introducing a score refinement module that extracts visual features of the skin and scores them using a Siamese network as a strategy during the training stage^3^. In another study, 18 skin diseases that exhibited erythema symptoms, including psoriasis, were classified by decomposing images into hemoglobin and melanin components from skin disease images as inputs for the EfficientNet model^9^. Furthermore, employing a CNN model facilitated the classification of psoriasis severity across individual lesions, encompassing the assessment of each severity for primary aspects: erythema, scaling, thickness, and affected area. A comparative analysis between the clinician’s disease assessment and the CNN model’s classification results highlighted the superior performance of the CNN-based lesion severity evaluation^19,20^. Previous studies have explored the classification (based on trained features) of various skin disorders, such as melanoma, dermatitis, and psoriasis^21,22^. However, most studies used only local images of individual conditions or overlooked the incorporation of multiple-severity disease categories in the evaluation of holistic disease images^23^. In this study, we propose a novel appoach to psoriasis assessment that accounts for multiple-severity disease analyses and aligns with established clinical evaluation criteria.

First, we generated multiple-severity images using CutMix, a data augmentation method. In recent classification model training, CutMix, which cuts and pastes patches in training images, has been widely used to improve the performance of deep-learning models^24^. In Cutout^25^, a portion of the image is cut and filled with zeroes, whereas in MixUp^26^, one image is superimposed onto another. In both cases, two images are linearly interpolated and combined such that the resulting image is indistinct and unnatural. In contrast, CutMix, which involves cutting out a portion of one image and pasting it as a patch onto another, is effective in classification tasks without casuing image data corruption^27^. In conventional CutMix, labeling is based on a higher proportion of patches within a mixed image. However, in our approach, we designate the region with higher severity as the representative region, which serves as the evaluation criterion for multiple-severity diseases and subseqeuntly label the mixed image accrodingly. We improve the accuracy of psoriasis severity evaluation by learning the various features of severe psoriasis using CutMix.

Next, we propose a self-attention module based on a deformable convolutional network (DCN)^28^. In the field of computer vision, visual attention is a method that learns the features of a region of interest in an image by extracting the correlation between the channel and spatial information within convolutional features. Self-attention can improve model performance with a small overhead and can be self-contained to be applied independently to various CNNs^17,29^. In the self-attention approach, a squeeze and excitation network (SENet)^30^, which extracts important features by assigning weights to each channel within the feature map and a convolutional block attention module (CBAM)^31^, which improves the performance using spatial information as well as channel information, are proposed. Recently, a multiscale channel attention module (MS-CAM) has been demonstrated to improve object recognition performance by extracting spatial and channel information across multiple scales^32^. In psoriasis, lesions, and noise, such as shadows and skin hair, are mixed; therefore, accurate detection of the lesion region affects the disease analysis performance. In particular, in cases of multiple-severity diseases, it is important to recognize the location and shape of the representative region accurately. To tackle these challenges, we have developed a multi-scale deformable attention module (MS-DAM) using DCNs, a convolutional method that dynamically detects irregular and complex patterns by adding an offset to the filter.

Our study proposes a novel evaluation method that considers the representative disease region, which is the clinical evaluation criterion, in the process of scoring PASI parameters and evaluating the severity of psoriasis. We enhanced the efficacy of psoriasis severity classification through the introduction of a CNN model incoporating the MS-DAM. This novel approach enabled accurate detection of the multi-scale and complex patterns inherent to psoriasis. Additionally, by training on a fresh set of multi-severity images generated via CutMix, the diversity of diseases increases, enabling a more robust severity features representation learning. We verified the representative region detection performance of the proposed MS-DAM and confirmed that it has superior performance compared to that of existing self-attention methods. In addition, we confirmed the improvement in the classification performance of the model by learning multiple-severity images generated using CutMix. Therefore, the proposed method represents an advanced approach that improves the accuracy and precision of psoriasis evaluation by effectively learning the different severity features of psoriasis.

## Results

**Figure 1:**
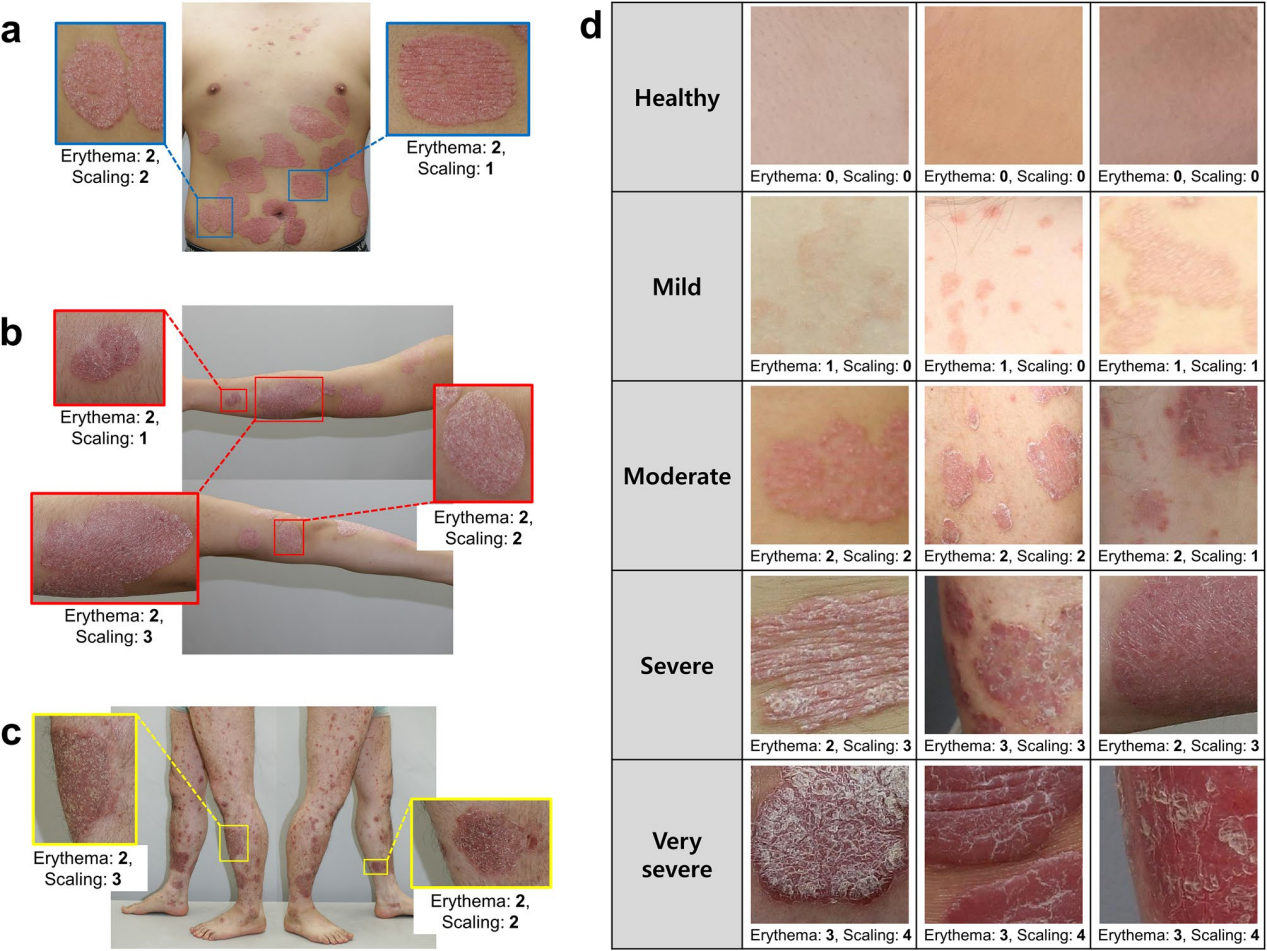
Local images of psoriasis in the five severity groups. We acquired local images of psoriasis distributed throughout the body and evaluated the PASI score for each local disease from a clinician. It was confirmed that psoriasis in the same area had different severity scores. (**a**) Trunk area. (**b**) Arm area. (**c**) Leg area. (**d**) Local psoriasis images, their erythema, and scaling scores in each severity group.

**Figure 2:**
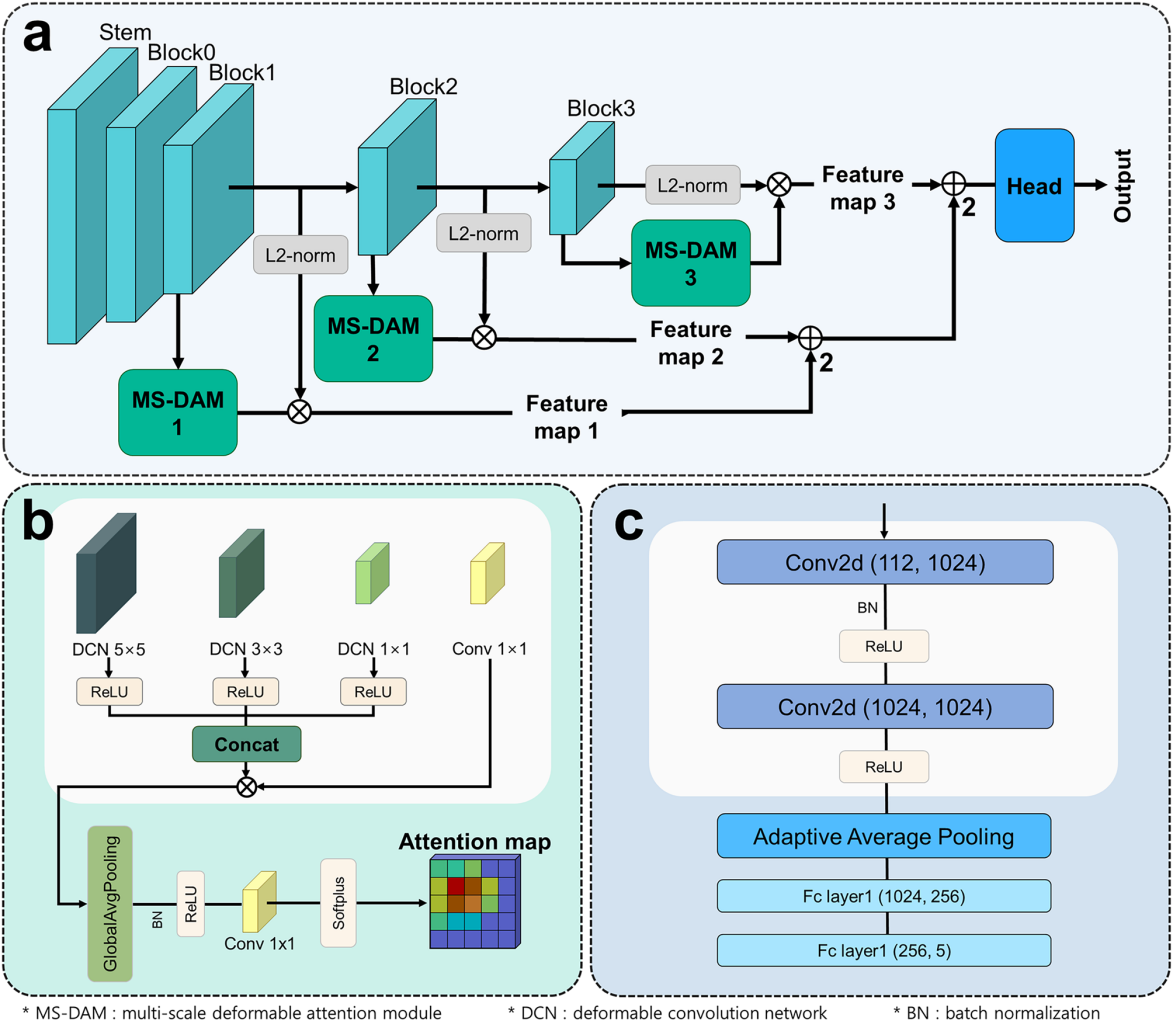
Overall architecture of severity classification using MS-DAM. (**a**) An end-to-end network that fuses hierarchical feature maps with MS-DAM and classifies severity. (**b**) Architecture of MS-DAM. (**c**) Architecture of the head.

We constructed a dataset of local disease images of the psoriasis area distributed throughout the body. For all experiment, informed consent was obtained from each patient and the tenets of the Declaration of Helsinki were followed throughout this study. Clinicians evaluated PASI scores for local disease images and divided the severity groups based on erythema and scaling scores (Fig. 1). In addition, we proposed the MS-DAM-based severity-classification network (Fig. 2).

### Validation of psoriasis severity classification by using MS-DAM

To verify the performance of psoriasis severity classification using the proposed self-attention module, MS-DAM, we used EfficientNet^33^ and RegNetY^34^ of SOTA models pretrained with ImageNet as backbone models for the classification task. We compared and analyzed the classification performance by grouping models with similar sizes and complexities. In addition, we trained the model on multiple-severity disease images using CutMix, with an execution probability, *P* of 0.3 and subsequently evaluated the classification performance of the model using the test dataset. The newly synthesized multiple-severity disease images were was labeled based on the representative area, irrespective of patch size. In other words, the proposed model learns the representation of a representative area of the multiple-severity disease images. Accuracy (ACC), precision (PR), recall (RE), and F1-score (F1) were used as evaluation indicators for model performance. Table 1 presents the classification outcomes of baseline models without the application of MS-DAM, alongside the classification results of the proposed method integrating MS-DAM, verifying its performance. Bold annotations in the table denote the best classification performance. Additionally, given the data imbalance issue in the psoriasis dataset used for experimentation, F1-score was adopted as the performance evaluation metric.

**Table 1:** Results of psoriasis severity classification using SOTA models.

Models	ACC (%)	PR	RE	F1	MACs(G)	Params(M)
(a) Baseline						
EfficientNet-B0	82.24	0.86	0.82	0.84	0.70	4.02
RegNetY-400MF	80.24	0.82	0.84	0.83	0.77	3.91
EfficientNet-B1	86.84	0.92	0.85	0.87	1.05	6.52
RegNetY-600MF	82.24	0.87	0.85	0.86	1.14	5.45
EfficientNet-B2	86.84	0.92	0.85	0.88	1.22	7.71
RegNetY-800MF	84.21	0.87	0.86	0.86	1.49	5.5
EfficientNet-B3	88.82	0.92	0.92	0.92	1.78	10.71
RegNetY-1.6GF	84.21	0.89	0.87	0.88	3.0	10.32
EfficientNet-B4	**89.47**	**0.93**	**0.92**	**0.92**	2.81	17.56
RegNetY-4.0GF	77.63	0.84	0.77	0.78	7.35	19.57
EfficientNet-B5	82.89	0.86	0.85	0.84	4.44	28.35
RegNetY-8.0GF	82.89	0.86	0.87	0.87	14.7	37.18
Avg. EfficientNet	86.18	0.90	0.87	0.88		
Avg. RegNetY	81.90	0.86	0.84	0.85		
(b) Using MS-DAM						
EfficientNet-B0	85.53	0.92	0.87	0.88	1.63	6.02
RegNetY-400MF	81.58	0.87	0.83	0.84	2.43	8.59
EfficientNet-B1	**90.79**	**0.94**	**0.92**	**0.93**	2.0	8.53
RegNetY-600MF	84.21	0.90	0.85	0.86	2.91	11.32
EfficientNet-B2	86.84	0.90	0.90	0.90	2.2	9.78
RegNetY-800MF	86.18	0.90	0.88	0.88	3.68	13.44
EfficientNet-B3	88.82	0.91	0.92	0.92	2.89	12.94
RegNetY-1.6GF	84.21	0.87	0.86	0.86	4.96	18.54
EfficientNet-B4	88.82	0.93	0.93	0.93	3.98	20.01
RegNetY-4.0GF	83.55	0.87	0.87	0.86	11.23	36.54
EfficientNet-B5	82.24	0.89	0.84	0.85	5.77	30.93
RegNetY-8.0GF	83.55	0.87	0.87	0.87	21.97	86.43
Avg. EfficientNet	87.17	0.92	0.90	0.90		
Avg. RegNetY	83.88	0.88	0.86	0.86		

(a) Classification results using the basic architecture of EfficientNet and RegNetY. (b) Classification results for both models using the proposed MS-DAM. Best performance values are in bold.

The classification results of the baseline models (Table 1a) show that EfficientNet exhibits superior performance over RegNetY. The average F1 value for EfficientNet was 0.88, whereas that of RegNetY was 0.85. In particular, the classification results of EfficientNet B4, which had the best performance, and that of its pair, RegNetY 4.0GF, showed a remarkable performance difference of 0.14. These performance differences were also apparent in models equipped with MS-DAM (Table 1b). The application of MS-DAM led to an average F1 value of 0.90 for EfficientNet and 0.86 for RegNetY, signifying EfficientNet’s superior classification performance. This suggests that EfficientNet is effective as a backbone model for psoriasis severity classification. In addition, comparing the performance of the baseline model and the proposed model with MS-DAM, the average F1 value improved by 0.02 in EfficientNet and 0.01 in RegNetY. Thus, MS-DAM proved effective in identifying discriminative differences in diseases according to severity and in improving the classification performance. However, in certain cases such as EfficientNet-B3, the performance difference between the baseline and MS-DAM-applied models was minimal. This variation in the impact of MS-DAM varies depending on the size and complexity of the model. Therefore, it is essential to select a suitable model for the data characteristics.

Additionally, to analyze the classification performance more accurately, both the PR and RE values should be considered. PR gauges the model’s consistency in maintaining good classification performance, while RE measures its effectiveness on real data. These two metrics, which exhibit a trade-off relationship, improve together for better model performance. With the integration of MS-DAM, both PR and RE values improved. Therefore, the proposed model detects subtle differences in disease severity and improves the precision and generalization performance of the model across various data processing tasks when compared to the baseline model. Futhermore, EfficientNet B1 demonstrated the highest classification performance when employing MS-DAM, boasting an F1 value of 0.93. It is worth noting that optimizing model efficiency by minimizing computational cost and power is also essential. EfficientNet B1, a lightweight model with a small size and less complexity, excelled in this aspect. Compared to RegNetY 800MF, the top-performing model in RegNetY series, EfficientNet B1 exhibited a superior performance with an ACC of 4.6% and F1 of 0.05 while being approximately 1.6 times smaller, thus ensuring high computational efficiency. In addition, comparing EfficientNet B4 from the baseline models with EfficientNet B1 applied with MS-DAM, the latter demonstrated the capacity to rapidly and effectively detect complex disease patterns within the lighter model. Therefore, MS-DAM-based fusion features are effective for classifying the severity of multiple diseases, and it is possible to evaluate psoriasis in a mobile environment using the proposed method.

**Figure 3:**
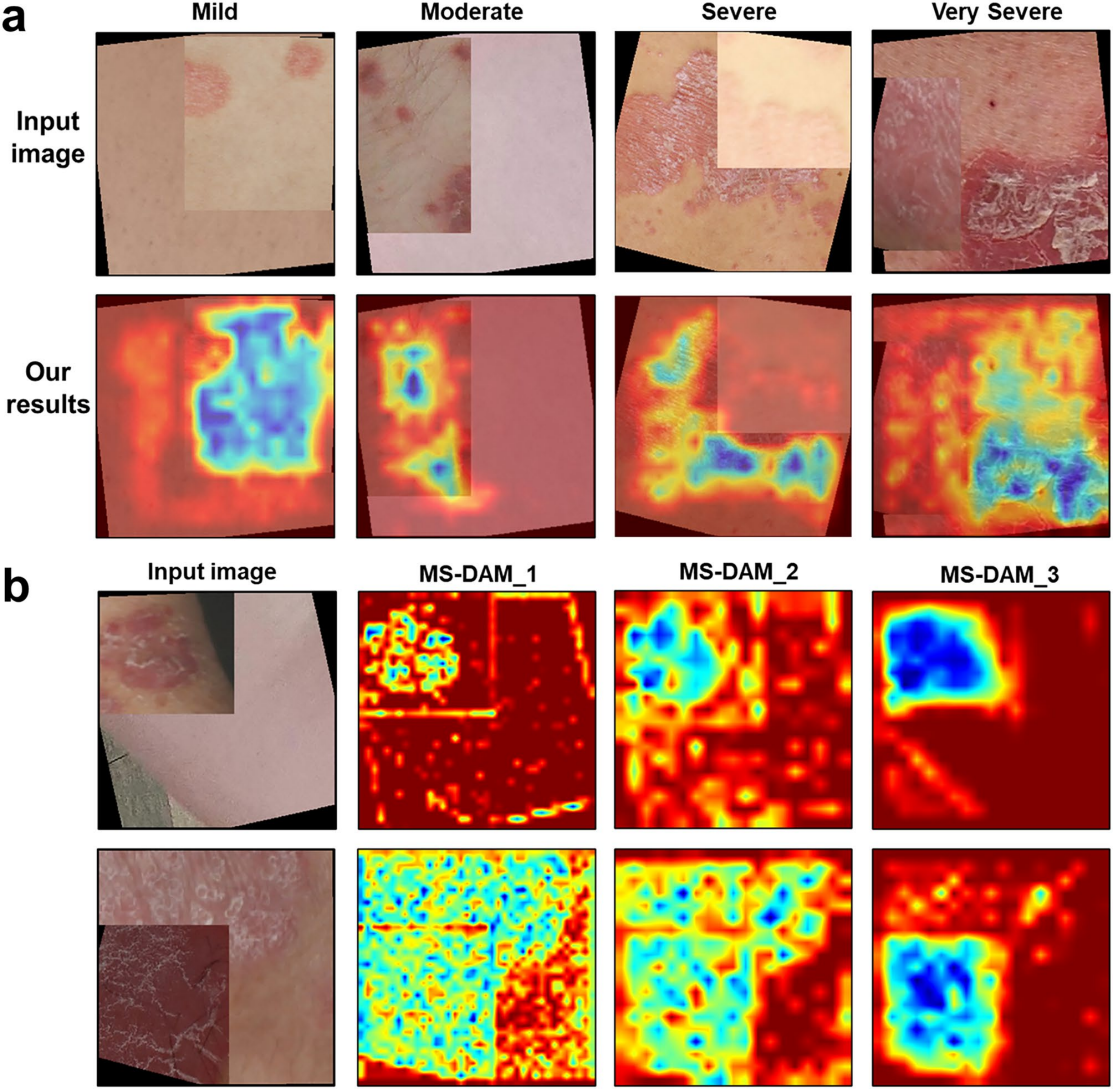
(**a**) Grad-CAM results of the attention region to which the proposed model pays attention in multiple-severity diseases of each severity group. (**b**) Hierarchical responses of feature maps with MS-DAM.

We used the gradient-weighted activation mapping method (Grad-CAM)^35^ to verify how the MS-DAM-applied model directs its attention towards multiple-severity disease images. We confirmed that the model accurately detected representative regions within multiple-severity disease images and observed that multiple MS-DAMs constituted hierarchical attention maps (Fig. 3). We employed EfficientNet B1, the most proficient model in the prior classification experiment, for visualization purposes. Figure 3a shows the attention regions the model focuses on within multiple-severity images according to severity groups. The model focuses on the region with higher severity, which is the representative region, across all the multiple-severity disease images. In images of severe and very severe groups, the model could distinguish between two different severity regions even if they were consecutively connected. In the moderate and severe group images, the skin color of the two merged images is different. Obeserving the results of Grad-CAM, we confirmed that the model pays more attention to the severity features rather than color difference. Figure 3b depicts the hierarchical attention responses from multiple MS-DAMs. As layers deepened, the extraction of semantic features from disease images was accurately depicted within the fusion feature map. The model recognized representative disease regions in multiple-severity diseases using the edge and texture features of the disease in the lower layers and the fusion semantic features in the deeper layers. In other words, the proposed method operated in harmony with human visual perception.

Overall, the generalized features of pre-trained models exhibited limitations in detecting psoriasis and learning severity features. Despite the intrinsic ambiguity of diseases and the presence of environmental noise, it is important to accurately detect the disease region to improve accuracy and precision. The adoption of the MS-DAM-based model enables more precise detection of disease areas and effective learning severity feature representation.

### Comparison with different self-attention methods

To evaluate the performance of the proposed MS-DAM, we compared and analyzed the classification results of the model with those of existing self-attention methods. The six existing self-attention methods include SENet, bottleneck attention module (BAM)^36^, CBAM, efficient channel attention for deep convolutional neural networks (ECA-Net)^37^, attention gated networks (AGUNet)^38^, and MS-CAM. Our method, MS-DAM, can be used as an independent modules module alongside these methods. Therefore, we conducted severity classification by applying each method to the hierarchical responses of EfficientNet B1, a backbone model. Similar to the previous experiment, we utilized CutMix with an execution probability of 0.3 for the training dataset, and evaluated model performance using the test dataset. Table 2 presents the classification performance for each self-attention method. Among the six existing methods, ECA-Net exhibited the best performance, achieving an F1 value of 0.91. ECA-Net is a self-attention method that improves the attention performance on an object by solving the dimensionality reduction problem, a drawback of SENet. ECA-Net reduces model complexity and introduces effective local cross-channel interaction. SENet followed closely with the second-best performance. Psoriasis severity is distinguishable through distinct color variations in erythema and scaling. Thus, ECA-Net and SENet improved the severity classification performance of the model by focusing on these color differences within the lesions. However, classification based solely on color differences can be challenging because of the cyclic stages of deterioration and improvement psoriasis undergoes. The color information of the lesion appears as a mixture across the image and varies with disease progression. Reliance solely on color informantion struggles to handle diseases with complex patterns and shapes. In contrast, our proposed MS-DAM yielded promising results with an F1 value of 0.02, suprassing ECA-Net. MS-DAM accurately identifies the attributes of representative disease regions—shape, size, and location—and extracts fine severity features by analyzing multi-scale features, thus showing good classification performance, particularly for challenging diseases with multiple-severity levels. Importantly, MS-DAM’s accuracy surpasses BAM by more than 7%, indicating a significant performance difference, and shows a performance advantage of over 5% compared to the latest method, MS-CAM. Therefore, the proposed MS-DAM exhibits better psoriasis severity detection and classification performance than other self-attention methods and is effective in learning image representations for multiple-severity diseases.

**Table 2:** Comparison of psoriasis severity classification performance according to the application of various self-attention methods.

Self-attention methods	ACC (%)	PR	RE	F1
SENet	86.18	0.88	0.90	0.89
BAM	83.55	0.88	0.88	0.88
CBAM	84.87	0.89	0.86	0.87
ECA-Net	**87.50**	**0.92**	**0.90**	**0.91**
AGUNet	86.18	0.89	0.90	0.89
MS-CAM	85.53	0.90	0.87	0.88
MS-DAM (ours)	**90.79**	**0.94**	**0.92**	**0.93**

Best performance values are in bold.

**Figure 4:**
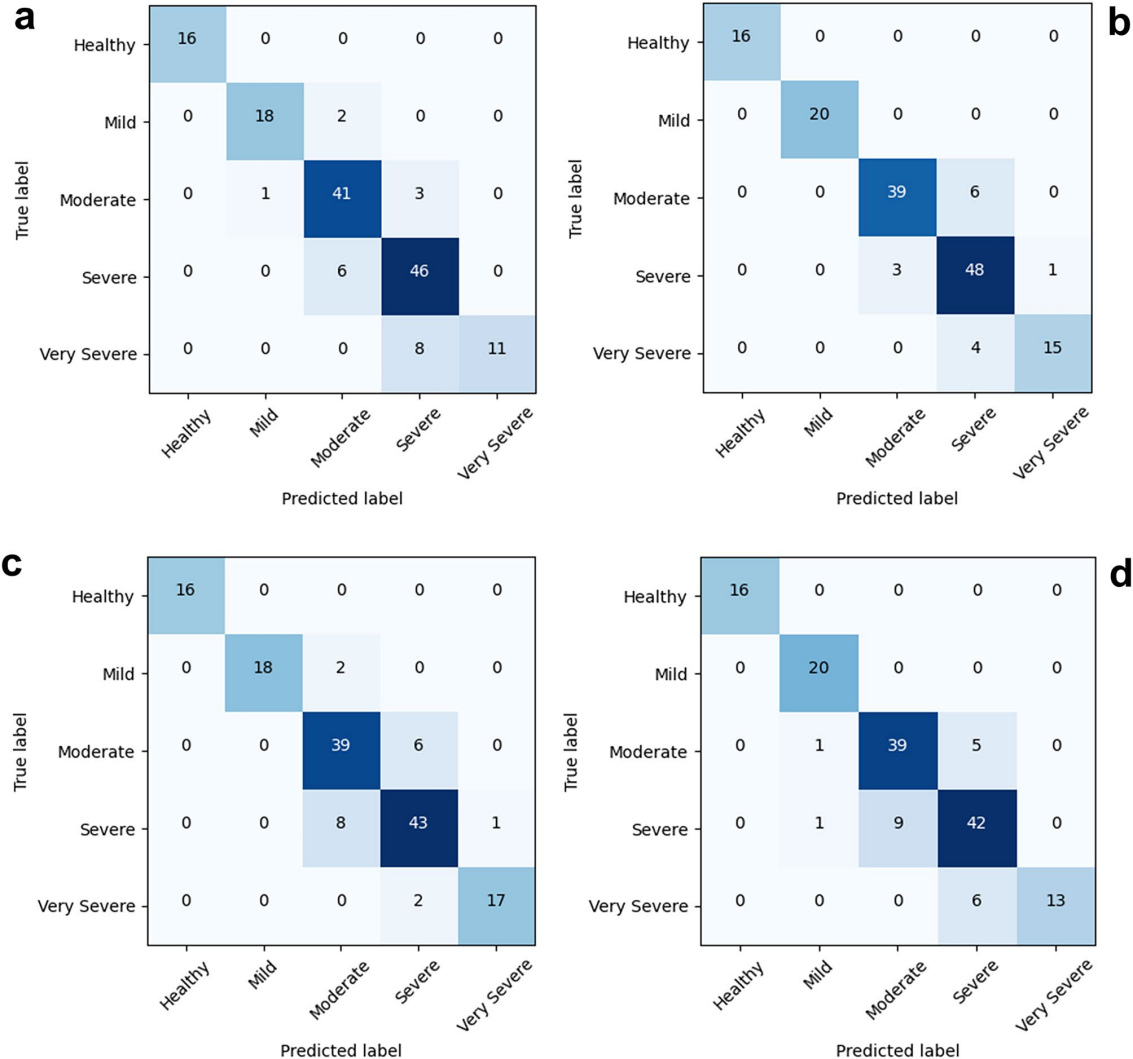
Confusion matrix according to the application of three self-attention modules in severity classification. The classification performance of the EfficientNet B1 (**a**) without self-attention module (baseline), (**b**) with MS-DAM, (**c**) with ECA-Net, and (**d**) with MS-CAM.

Figure 4 shows the confusion matrix depicting psoriasis detection performance when utilizing ECA-Net (the most effective among the existing methods), MS-CAM (the latest method), and our proposed MS-DAM. Figure 4a illustrates the results of sequential application starting from the EfficientNet B1-baseline (a) and progressing to MS-DAM (b), ECA-Net (c), and MS-CAM (d). In the baseline result (a), all severity groups except the healthy group underwent misclassification into neighboring groups. Notably, the highest misclassification was observed within the very severe group. Suprisingly, severe and very severe group images were frequently identified as lower severity groups. This misclassification stems from the high disease similarity between adjacent groups, with features of the moderate and severe groups, more extensively trained with data, predominantly influencing the process. In the MS-DAM enhanced confusion matrix (b), although misclassification from moderate to severe group increased slightly, errors in the opposite direction (severe to moderate, very severe to severe) diminished. With reference to the results in Table 1, despite class imbalance, the model presented in (b) adeptly classified diseases across groups compared to the baseline model in (a), leading to an improvement in the four classification indicators. Notably, the increased Recall (RE) value signifies enhanced model robustness in real-world disease evaluation. The confusion matrices (c) and (d) for remaining two self-attention methods displayed a reduction in misclassification within the very severe group; however, misclassification issues persisted within the moderate and severe groups. Given the complex and dynamic features of diseases, misclassification concerns are inevitable. Thus, leveraging attention features collectively can mitigate disease evaluation errors by accurately identifying disease shapes, patterns, and training severity features more effectively.

**Figure 5:**
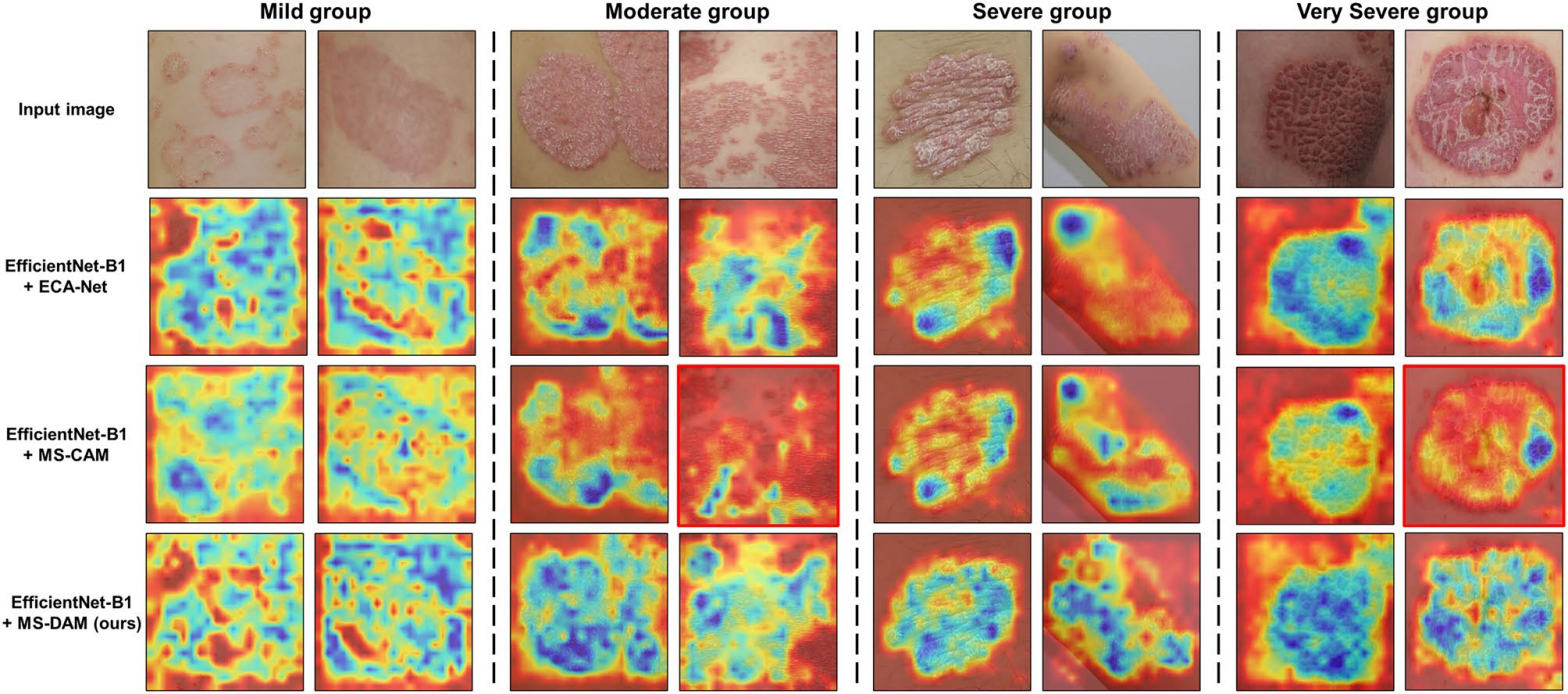
Grad-CAM results according to the application of the three self-attention modules in severity classification. The proposed MS-DAM accurately detects disease regions regardless of the shape, size, and location.

Additionally, in Fig. 5, we employed Grad-CAM to visualize the responses of the aforementioned three self-attention methods to actual severity images. In Grad-CAM results, the blue regions denote areas deemed significant by the model in severity classification. A close alignment between the blue area and the disease area indicates enhanced disease detection performance of the model. Images depicting mild severity disease showed similarities on color and texture between lesions and surrounding healthy skin. As a result, the model detected the entire area rather than focusing on the specific affected region. In images from severe or very severe groups, clear differentiation existed between the disease area and the healthy skin, prompting the model to detect semantic information such as disease shape and location. Within MS-CAM, images highlighted with red boxes denote misclassification results. Based on these, MS-CAM failed to recognize disease areas (moderate group) or solely detected a partial area (very severe group). Comparatively, ECA-Net accurately classified representative images from the four groups; however, compared to MS-DAM results, discrepancies in the focused area (blue region) were evident in moderate and severe groups images. In the case of second disease within the moderate group, MS-DAM more accurately identified the disease area, whereas ECA-Net failed to recognize the densely populated region of smaller diseases and the boundary of the affected area. Moreover, in the second disease within the severe group, ECA-Net’s focuse on the shaded area of the highly curved elbow differed from wide-spreading disease area recognized by MS-DAM.

**Figure 6:**
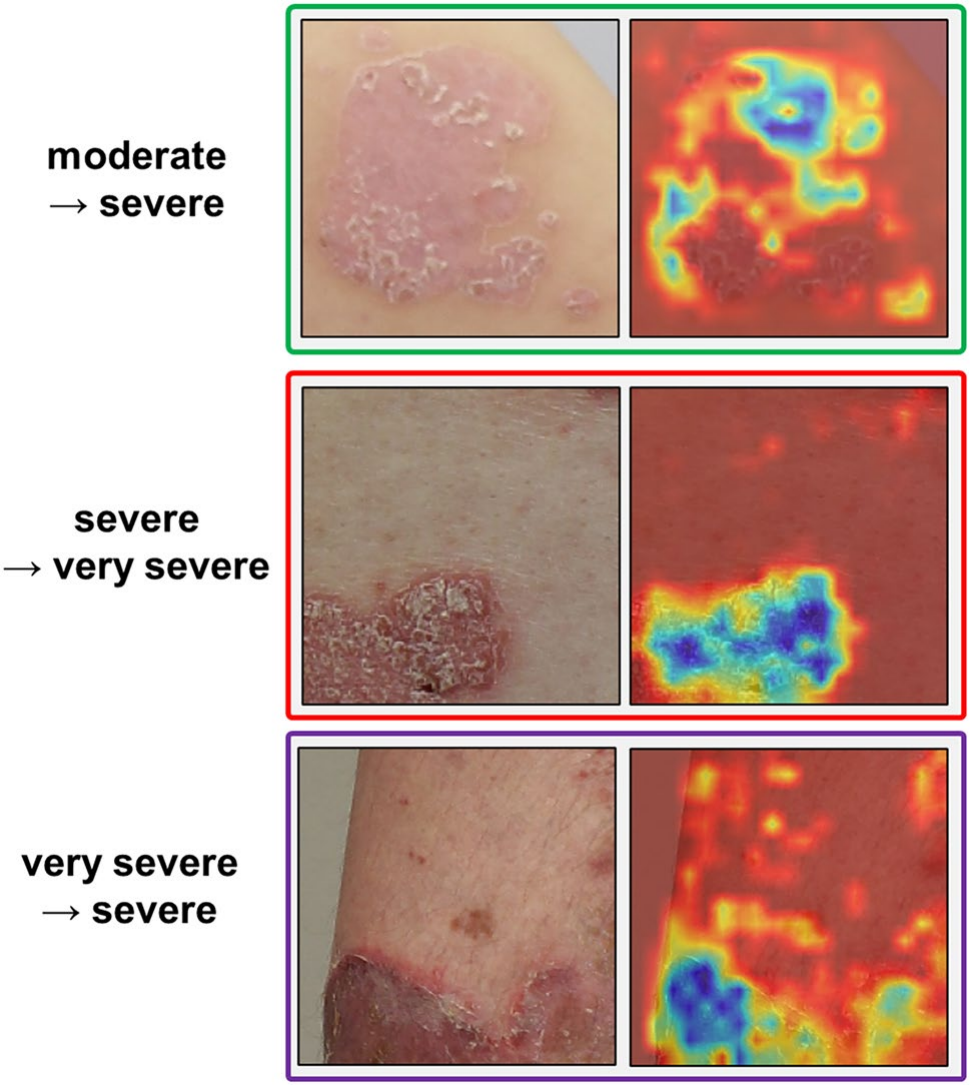
Misclassification cases. We highlight misclassification cases for the moderate, severe, and very severe group images of the proposed model.

Lastly, Fig. 6 displays the misclassification results of the proposed MS-DAM-based model using Grad-CAM. In instances where an image from the moderate group was misclassified as severe, the model concentrated on the upper part of the less affected region. This points to a challenge in the model accurately detecting disease shape and area. Conversely, misclassifications in the severe and very severe groups showcased precise disease area detection. Within the severe group image, erythema and scaling interwined in the detected disease area, reflecting characteristics similar to the very severe group and leading to misclassification. The phenomenon arises from the varying prominence of lesions, forms, and patterns across patients and severity levels, combined with the high similarity of lesions between severity groups.

### Results on CutMix execution probability

**Figure 7:**
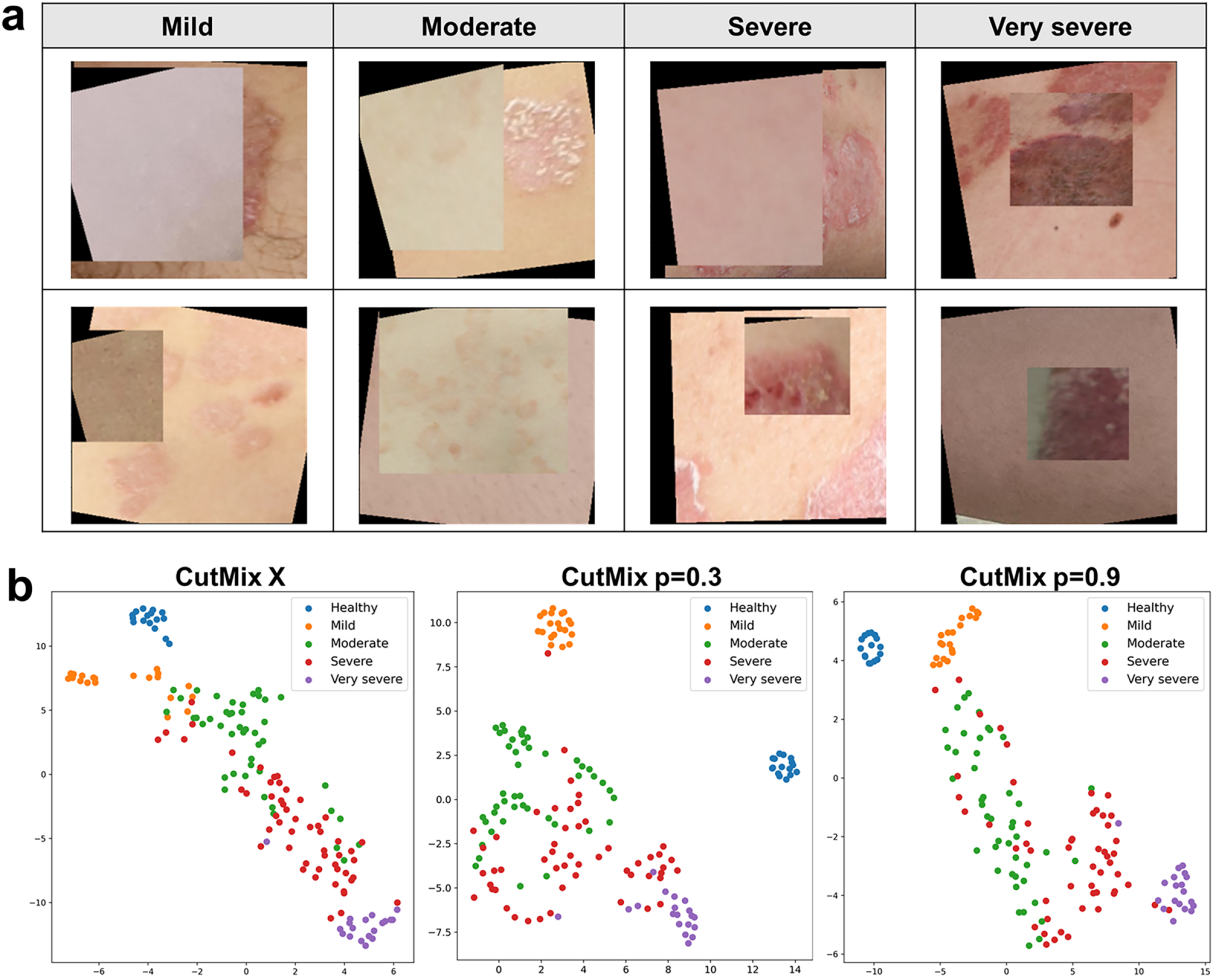
(**a**) Multiple-severity disease images generated using the CutMix. (**b**) Distributions of learning features by severity group according to the CutMix execution probability.

We applied geometric transformation and CutMix as data augmentation techniques to diversify the training dataset of disease images. Synthesized images combining two distinct severity images, randomly selected from the entire group, held a higher probability of reflecting elevated severity due to labeling criteria. Through comprehensive learning of diverse diseases, the issue of ambiguous lesion features arising from dynamic changes in psoriasis was reduced, and the generalization of the model was improved. In addition, the precision of the model was improved because the model’s learning criteria for multiple-severity diseases aligned with actual clinical evaluation procedures, mirroring human visual perception. Figure 7a illustrates the creation of novel multiple-severity diseases achieved by simultaneously applying geometric transformations and CutMix. To prevent biased feature learning from the freshly synthesized images, we explored the optimal criterion for improving model performance by adjusting the CutMix execution probability. Table 3 presents the classification outcomes based on the execution probability value *P* of CutMix. We used EfficientNet B1 with MS-DAM, which yielded the best performance in prior results, we found that when *P* was set at 0.3, the classification result was optimal, yielding an F1 value of 0.93. Compared to the CutMix-absent result (*P* = 0), the introduction of CutMix (*P* = 0.3) improved the ACC by over 7% and F1 value by 0.7. Additionally, in all instances of the CutMix application (*P* = 0.1–0.9), classification performance outperformed that of non-CutMix scenarios. Furthermore, when setting *P* between 0.2 and 0.4, the diversity of data increased, and superior performance was achieved. We confirmed that CutMix improves the robustness of the model by learning various severe psoriasis images and presenting the optimal execution probability that improves model performance.

**Table 3:** Comparison of psoriasis severity classification results according to CutMix application rate.

Probability	ACC (%)	PR	RE	F1
0.0	83.55	0.90	0.84	0.86
0.1	86.84	0.92	0.89	0.90
0.2	88.82	0.94	0.88	0.90
0.3	**90.79**	**0.94**	**0.92**	**0.93**
0.4	88.16	0.92	0.89	0.90
0.5	83.55	0.87	0.88	0.87
0.6	86.18	0.90	0.91	0.90
0.7	84.87	0.89	0.90	0.89
0.8	84.21	0.89	0.89	0.89
0.9	88.16	0.91	0.92	0.91

Best performance values are in bold.

We conducted a through comparison and analysis of feature distributions across severity groups based on various CutMix execution probabilities (*P* value). Figure 7b displays distribution graphs of learned features for *P* values 0, 0.3, and 0.9. Initially, when CutMix was absent (*P* = 0), features of adjacent severity groups demonstrated close distribution, ecepting the healthy group. This phenomenon stems from the comprehensive assessment of psoriasis across diverse lesions, masking it challenging to pinpoint unique features specific to each severity group due to varying dominant lesionsamong diseases. Moreover, given psoriasis’ chronic nature, characterized by cyclical improvements and deteriorations, dynamic disease changes can be detected. The intricate, continuous patterns in psoriasis lead to eventual similarity and ambiguity between severities. Consequently, even in the moderate and severe groups, where substantial disease data are available, features learned for both groups tend to merge. Conversely, the limited number of images for severe disease scenarios complicates the discriminative features learning for each severity group, resulting in misclassification within neighboring severity groups. At an execution probability of 0.9, moderate and severe group features appeared intertwined in distribution. A high ratio of new multiple-severity images in the training dataset can excessively alter the distribution of inherent features tied to actual severity. However, CutMix application inclines to generate high severity multiple-severity images, as demonstrated by the clearer separation of the very severe group distribution at an execution probability of 0.9. Hence, multi-severity images effectively enhance disease representation learning for the very severe group. At an the execution probability of 0.3, feature distribution for each group displayed greater distinctiveness than the other cases. Notably, the application of CutMix resulted in distinct regions for mild group features. These findings confirm the optimal execution probability of CutMix, which enhanced model feature representation capabilities and maximizing performance.

### Ablation study

We conducted an ablation study to ascertain the impact of three training settings, CutMix, MS-DAM, and the focal loss function, on overall performance. These three factors were selected as ab optimal combination for accurate and effective detection of various disease patterns. Table 4 showcases classification performance for the baseline scenario without all three factors and three cases incorporating each factor. Compared to the baseline and each individual factor, incorporating the focal loss function yielded improvements across PR, RE, and F1 indicators. This function enhances disease detection performance by effectively distinguishing afflicted areas from healthy ones. Subsequently, utilizing CutMix alongside geometric transformations as data augmentation improved indicators other than PR. CutMix-generated data may slightly perplex the model due to the ambiguity of diseases characterized by irregular and dynamic features. However, it effectively enhances model robustness and generalization by training on challenging data that adheres to actual labeling criteria. Finally, models incoporating MS-DAM exhibited the most significant performance enhancement, with an F1 value increase of 0.5 compared to the baseline outcome. This outcome validates the effectiveness of multi-scale attention features in extracting and learning disease features. By meticulously comparing and analyzing performance across the three training settings, we affirm the collective significance of all three factors in improving disease detection performance.

**Table 4:** Comparison of psoriasis severity classification performance according to the three training settings.

Setting	ACC (%)	PR	RE	F1
Baseline				
Only backbone	83.55	0.88	0.83	0.85
+ *Cross entropy*				
+ *Geometric transform*				
Loss function				
Focal loss	83.55	0.89	0.85	0.87
+ *Geometric transform*				
+ *Only backbone*				
Data Aug				
CutMix	84.87	0.86	0.89	0.87
+ *Cross entropy*				
+ *Only backbone*				
Self-attention				
With MS-DAM	88.16	0.91	0.89	0.90
+ *Cross entropy*				
+ *Geometric transform*				

## Discussions

Psoriasis, a chronic skin ailment, exhibits recurring cycles of improvement and deterioration during treatment process. This treatment course prompts dynamic changes in lesion features, such as shape, size, texture, and color, leading to varying degrees of disease severitys. Accurate evaluation of multiple-severity disease is challenging owing to the ambiguity of the disease severity; hence, studies that reflect and evaluate the multiple-severity of the disease remain scarce. Our study proposed a psoriasis severity classification method that considers the irregular features of psoriasis and clinician-based evaluation criteria. First, we generated multiple-severity disease images using CutMix, addressing limited dataset concerns while enhancing generalization performance of the model. In addition, we proposed hierarchical MS-DAMs to detect the representative region and identify severity features in multiple-severity diseases. The proposed MS-DAM emphasizes small-scale objects within local areas and extends its focus to larger objects distributed throughout the entire region, using fused multi-scale features to detect sectional and localized features. Thus, this is effective in detecting the representative region, which is an essential severity evaluation criterion and has various sizes, shapes, and locations.

First, we compared the classification results of the baseline models of the basic model architecture and those of the models applying MS-DAM. When MS-DAM was employed, we observed a modest average F1 enhancement of approximately 0.02 for both EfficientNet and RegNetY. Given psoriasis’s intricate lesion ambiguity across severity groups, it is important to improve the precision of the model, which defines the performance of the model in identifying lesion features. Although it is a minimal performance improvement, the application of MS-DAM improved both PR and RE, thereby improving the robustness of the model. In terms of analyzing psoriasis severity, EfficientNet outperformed RegNetY. EfficientNet B1 with MS-DAM exhibited superior performance, with an F1 of 0.93. This result underscores the potential for psoriasis diagnosis in mobile environments via the lightweight model (EfficientNet B1) with its compact size and low complexity. We then assessed the performance of the proposed MS-DAM using Grad-CAM. In addition, the detection performance of the representative region was validated for mixed severity images. Consequently, we confirmed that the proposed model accurately identified a representative region, regardless of size, shape, and location. Futhermore, for the hierarchical attention responses of multiple MS-DAMs, we confirmed that, by fusing multi-scale deformable attention maps, the model detects disease regions similar to human visual perception procedures.

Next, we compared and analyzed MS-DAM’s the performance against six estalished self-attention methods. We used SENet, BAM, CBAM, ECA-Net, AGUNet, and MS-CAM. Leveraging these as independent modules, we applied each self-attention method to EfficientNet B1, which previously exhibited the best performance results and compared the classification results accordingly. MS-DAM demonstrated superior performance compared to that of other self-attention methods. While existing self-attention techniques amplify object identification performance by emphasizing the dominant channel and spatial features, psoriasis, distinguised by color discrepancies between erythema and scaling, experiences color feature ambiguity as the disease progresses through stages. Thus, the color features of the disease minimally impacted the performance improvement of the model. The proposed MS-DAM robustly identifies the representative region even with various geometric image variations and extracts multi-scale severity features, and accurately determines the severity with enhanced reliability.

Lastly, we evaluated the efficacy of learning multiple-severity disease representations using CutMix on model performance. The creation of multiple-severity disease images generated using CutMix addressed data paucity concerns in severe psoriasis groups notably the very severe group. Additionally, by encompassing various disease severity types, the model’s generalization performance can be improved. We determined the optimal CutMix execution probability by adjusting it from 0.1 to 0.9. When CutMix was applied with a probability of 0.3, the accuracy improved by more than 7% compared to that when CutMix was not used. In the distribution of learning features by class according to execution probability, the ambiguity of features between adjacent groups of severity was alleviated by applying CutMix.

The proposed method learned severity feature representations and detected the distribution region of the disease by reflecting a clinician’s visual inspection procedure to accurately evaluate various types of psoriasis, such as multiple severity diseases. This could overcome the ambiguity of severity attributes in chronic skin conditions and facilitate precise psoriasis assessment in line with real inspection criteria.

We proposed a novel method for assessing psoriasis; however, certain challenges persist. The lack of consideration for variations in skin color emerges as a limitation because of our exclusive use of data from Korean psoriasis patients in the experiment. Environmental factors such as lighting conditions during image capture can introduce variations in skin color. To address this, future work will involve enhancing our approach by analyzing the impact of environmental noise like shadows, reflections, and divers skin tones on psoriasis assessment. Furthermore, we aim to extend our proposed model’s applicability to other skin conditions, such as atopic dermatitis, exhibiting various degrees of severity similar to psoriasis. To bolster the model’s versatility, we paln to validate its performance using open datasets that encompass different factors like ethnicity, and also engage in a comparative analysis with existing psoriasis assessment methods.

## Conclusion

Psoriasis is a chronic skin disease characterized by repeated deterioration and requires treatment for months or even years. Evaluating the severity of psoriasis typically relies on visual inspection-based metrics like the PASI score. Psoriasis exhibits dynamic changes as lesions develop, deteriorate, or disappear during treatment. With these dynamic changes, multiple-severity diseases are observed within the diagnosis range. This diversity in severity necessitates subjective clinician-driven selection of representative areas for evaluation. However, this approach often leads to discrepancies in clinical indicator assessments. Thus, objectifying and quantifying the PASI scores based on straightforward clinical labels prove challenging. Our research addresses this need by proposing a psoriasis evaluation approach that aligns with the clinician’s perspective. This approach involves the generation of diverse-severity disease images using CutMix to increase the diversity of the training data. Our proposed MS-DAM method is a self-attention method that can accurately detect a representative region of the disease to perform psoriasis severity classification reflecting the clinical evaluation perspective. Furthermore, it detects an evaluation region of a disease with irregular size and shape and complex patterns by fusing multiscale deformable attention maps. The deformable attention features extracted from the hierarchical information of the model contain spatial and textural information of the disease. Through comparisons with baseline models, we highlight that incorporating MS-DAM into EfficientNet B1 yields superior classification performance. Moreover, we verified that the proposed MS-DAM is more effective for psoriasis severity classification than six existing self-attention methods. Lastly, we presented the utility and optimal learning rate for multiple-severity datasets of training data through performance comparison and analysis according to the execution probability of CutMix. When the execution probability was set to 0.3, the features of each severity group were more clearly distinguished by training various severe disease images, and the classification performance and generalization of the model were improved. The proposed method can be used to evaluate various skin disease and is expected to be used as a method to increase the precision of the model by reflecting a clinician’s subjective evaluation criterion. Furthermore, MS-DAM can be applied to various tasks, such as the segmentation and prediction of medical images.

## Methods

The proposed method is an end-to-end network that learns feature extraction using MS-DAM, multi-scale feature map fusion, and severity classification processes. Figure 2a shows the overall network architecture that fuses hierarchical attention maps extracted using multiple MS-DAMs and classifies psoriasis severity through the head part. Figure 2b shows the architecture of MS-DAM, and Fig. 2c shows the architecture of the head.

### Datasets

We acquired images of psoriasis from 44 Korean patients at the Department of Dermatology, Korea University Guro Hospital. All subjects participated voluntarily and provided their written informed consent to participate in this study. The participating institutions, namely the Korea University Guro Hospital and Soonchunhyang University, conducted this study in accordance with the ethical standards of Helsinki Declaration, ICH-GCP standards and follwed all applicable regulations (Approval number: 2020GR0019, 202001-BM-005). In addition, all methods of the experiment were performed in accordance with the relevant guidelines and regulations, such as anonymization of personal information.

We constructed a dataset from the local psoriasis images containing one disease area^18^. Local images of psoriasis were cropped into four or six parts based on the sizes of the images, and the cropped images were resized to 300 × 300 pixels. Additionally, the severity of psoriasis was labeled based on erythema and scaling scores, which are the evaluation factors of the PASI score. A dermatologist assigned zero to four points to local psoriasis images according to the severity of erythema and scaling (0, healthy; 1, mild; 2, moderate; 3, severe; 4, very severe). We divided the psoriasis severity into five levels based on the sum of the scores of the two evaluation factors. The five groups were healthy (0 points), mild (1–2 points), moderate (3–4 points), severe (5–6 points), and very severe (7–8 points). Figure 1 presents local images of psoriasis according to its severity. The entire data was divided based on the patient so that training and test data didn’t overlap. We used 640 training images (healthy (0): 41, mild (1): 141, moderate (2): 186, severe (3): 206, very severe (4): 66) and 152 test images (healthy (0): 16, mild (1): 20, moderate (2): 45, severe (3): 52, very severe (4): 19) in the experiment.

### Data augmentation

We used two data augmentation methods to prevent overfitting of the model and improve the performance by increasing the amount of data. First, we applied the general augmentation method, which uses geometric transforms, such as rotation [− 15, 15] and vertical/horizontal flip. Next, we used the CutMix method. CutMix is a cut-and-paste method that cuts part of an image and fills it with a patch from another image^27^. This can improve the generalization and localization performance of a model by allowing it to learn the less important part and the entire image, rather than focusing only on important features within an image.

Psoriasis presents diverse morphological attributes, with lesions exhibiting varying shape and size, and irregular complexities. However, the severity score of the lesion was evaluated without accounting for this irregularity. Futhermore, assessing the severity of conditions with multiple gradations, characterized by ambiguous lesions, remains a challege. Clinicians, in their evaluation, designate a representative region for multiple-severity cases, introducing subjectivity and consequential deviations in assessment outcomes. In a bid for more accurate severity evaluations, an approach that encapsulates the spectrum of severe disease patterns and clinical evaluation critera is indispensable. Regrettably, most studies focusing on psoriasis assessment diregard the clinical perspective. Our research addresses this gap by leveraging CutMix to generate a fresh array of diverse-severity images. By labeling these images based on higher-severity patches, regardless of their spatial distribution or size, we attempt to align with the clinician defined criteria. In tandem, clinicians validate the newly generated images and their labeling criteria. By training models on merged disease images, we tackle data scarcity and expand our grasp on various disease characteristics. Furthermore, this approach mitigates data scarcity in the very severe group, enhancing robustness through an exposure to an array of severity variations. The following equations define the CutMix formula for generating a multiple-severiy disease image:

1 $$\begin{aligned} \tilde{x} & = M \odot x_{A} + \left( {1 - M} \right) \odot x_{B} \\ \tilde{y} & = \left\{ {\begin{array}{*{20}l} {y_{A} ,} \hfill & {if y_{A} > y_{B} } \hfill \\ {y_{B} ,} \hfill & {else} \hfill \\ \end{array} } \right. \\ \end{aligned}$$

A new image ($\tilde{x}, \tilde{y}$) is generated from two images and labels $(x_{A} , y_{A} ), \left( {x_{B} , y_{B} } \right)$. M denotes the mask area in which the images are combined. Here, M is set to a random value. In addition, we set the execution probability of CutMix from 0.1 to 0.9, and compared and analyzed the optimal condition.

### Multi-scale deformable attention module

We propose a multi-scale deformable attention module (MS-DAM) to detect representative regions and identify severity features accurately in multiple-severity diseases with irregular and complex patterns. Spatial features, such as shape, texture, and location of psoriasis within the observation area are irregular and complex. In convolution, a filter of a fixed size and shape extracts features that have limited spatial information, i.e., local information in a fixed receptive field. The use of single-size filters is also limited in the extraction of various hierarchical representations of convolutional networks^39,40^. In addition, for accurate evaluation of psoriasis diseases, including multiple-severity diseases, severity features such as texture information of scaling and semantic information on the representative region should be simultaneously extracted. Therefore, we used multi-scale DCNs^28^ to extract discriminative features for psoriasis according to the severity and learn the effective semantic information of the representative region. A DCN is a method that can robustly detect objects despite geometrical deformations such as scale, pose, and viewpoint by adjusting the offset, which is the convolution sampling location. The DCN can flexibly extract irregular and complex features within the receptive field.

In Fig. 2b, MS-DAM concatenates the feature maps of multi-scale DCNs with three kernel sizes and performs a residual connection using a 1 × 1 convolution layer to prevent gradient-vanishing and preserve hierarchical information. When the kernel size is small, low-frequency information, such as edges, can be extracted, and when the kernel size is large, high-frequency information can be extracted. Both low- and high-frequency information are important for learning disease representations according to the severity^40^. Then, multi-scale features undergo global average pooling, which reduces parameters while maintaining feature nonlinearity, a 1 × 1 convolution layer for feature normalization, and a softplus activation function. Finally, an attention map of irregular object features was generated. The hierarchical features are normalized using the L2-norm, and the final feature map is generated element-wise with the attention map. In addition, in the lower layer of the CNN, low-level components, such as the edges and contours of the image can be detected, and more complex patterns can be detected in the deeper layer. We obtained hierarchical feature maps using MS-DAM, fused them, and generated the final feature map. Therefore, the proposed method can clearly identify the semantic features of the representative region of the disease and detect fine differences in severity, that is, local features, by extracting and fusing multi-scale features that respond sensitively to various image information and dynamic changes in psoriasis.

### Training and evaluation strategies

Training and testing were performed using Tensorflow (backend Keras) and GeForce RTX 2080 Ti 11 GB GPUs (× 2). To verify the effectiveness of the proposed attention module, we used EfficentNet and RegNetY as backbone models, which are SOTA models for image recognition tasks. RegNetY is a convolutional network with a simple and regular design space that represents effective parameters, such as depth, initial width, and gradient of the model. EfficentNet is a convolutional network that can efficiently adjust the depth, width, and input size of a model using a compound scaling method. The two models were pre-trained with ImageNet and fine-tuned using our dataset. In addition, the outputs of stages 2, 3, and 4 of each model were used as the inputs of the attention module. We compared and analyzed the performance of two models with similar sizes and complexities.

The presence of class imbalance within our dataset prompted us to implement focal loss, a cost-sensitive learning method, as our loss function. Cost-sensitive learning is used to solve the class imbalance problem by assigining higher weights for a small amount of data. Focal loss is a loss function that weights higher for complex or easily misclassified data points^41,42^. In our case, by adjusted parameters $\alpha =0, \beta =1$ to adjust the overall loss value. The intricacies of multiple-severity disease images can lead to misclassification owing to overlapping severity attributes, amplifying confusion during the evaluation. The application of focal loss is our strategy to tackle class imbalance and address these intricacies more effectively. We adopted the rectified Adam (RAdam)^43^ optimizer and a cosine learning rate decay scheduling, with the learning rate initiated at 0.000001 and capped at 0.002. A training epoch of 50 coupled with a batch size was set of 16 was empirically established, considering convergence of training accuracy and loss metrics. To avert model overfitting, early stopping was employed, with the threshold set at 15.

Our model evaluation relied on metrics such as overall accuracy, macro-average precision, recall, and F1-score. Furthermore, we compared and analyzed the model performance by considering the model complexity and computational efficiency using parameteres such as model params and multiply-accumulate operations (MACs). Model params account for the parameter storage cost, whereas MACs represent arithmetic operations like multiplication and addition.

### Visualization

Class activation mapping serves as a fundamental technique for explainable artificial intelligence (AI), illustrating regions the model deems significant in an image for a given class. To elucidate and visually interpret our model’s severity feature representation, we employed the Grad-CAM visualization technique^35^.

### Ethical approval and consent to participate

Ethics approval for the use of data was granted by two institutional review boards (Approval number: 2020GR0019, 202001-BM-005), and informed patient consent was waived.

## Data Availability

The datasets generated and analysed during the current study are not publicly available due to restrictions in the data-sharing agreement but are available from the corresponding author on reasonable request.
